# Wood Litter Consumption by three Species of *Nasutitermes* Termites in an Area of the Atlantic Coastal Forest in Northeastern Brazil

**DOI:** 10.1673/031.010.7201

**Published:** 2010-06-22

**Authors:** Alexandre Vasconcellos, Flávia Maria da Silva Moura

**Affiliations:** ^1^ Departamento de Botânica, Ecologia e Zoologia, Centro de Biociências, Universidade Federal do Rio Grande do Norte, 59072-970, Natal, RN, Brazil; ^2^ Departamento de Systemática e Ecologia, Centro de Ciências Exatas e da Natureza, Universidade Federal da Paraíba, 58051 -900, João Pessoa, PB, Brazil

**Keywords:** abundance, biomass, consumption rates, decomposition, Isoptera, Neotropical region

## Abstract

Termites constitute a considerable fraction of the animal biomass in tropical forest, but little quantitative data are available that indicates their importance in the processes of wood decomposition. This study evaluated the participation of *Nasutitermes corniger* (Motschulsky) (Isoptera: Termitidae), *N. ephratae* (Holmgren), and *N. macrocephalus* (Silvestri) in the consumption of the wood litter in a remnant area of Atlantic Coastal Forest in northeastern Brazil. The populations of this species were quantified in nests and in decomposing tree trunks, while the rate of wood consumption was determined in the laboratory using wood test-blocks of *Clitoria fairchildiana* Howard (Fabales: Fabaceae), *Cecropia* sp. (Urticales: Cecropiaceae), and *Protium heptaphyllum* (Aublet) Marchand (Sapindales: Burseraceae). The abundance of the three species of termites varied from 40.8 to 462.2 individuals/m^2^. The average dry wood consumption for the three species was 9.4 mg/g of termites (fresh weight)/day, with *N. macrocephalus* demonstrating the greatest consumption (12.1 mg/g of termite (fresh weight)/day). Wood consumption by the three species of *Nasutitermes* was estimated to be 66.9 kg of dry wood /ha/year, corresponding to approximately 2.9% of the annual production of wood-litter in the study area. This consumption, together with that of the other 18 exclusively wood-feeders termite species known to occur in the area, indicates the important participation of termites in removing wood-litter within the Atlantic Coastal Forest domain.

## Introduction

Tropical forests and savannas are among the biomes that retain the greatest global diversity and abundance of termites (Lee and Wood 1971; Martius 1994; Bignell and Eggleton 2000). The biomass of termites in these ecosystems can be greater than 100 kg (fresh weight) per hectare (Wood and Sands 1978; Abe and Matsumoto 1979; Eggleton et al. 1996), which is much larger than the biomass of many groups of vertebrates in the Amazon Forest (Fittkau and Klinge 1973) or in the savannas of Africa (Deshmukh 1989).

Termites feed on a diverse range of resources, including live and dead wood, litter, humus, lichens, fungi, grass, manure, and animal corpses (Wood 1978; Noirot 1992). Wood consumption by these insects is apparently determined by properties related to their ability to masticate, digest, and assimilate it. As such, wood properties such as density, nitrogen concentration, the presence of phenols and quinones, and its level of decomposition, can all affect consumption rates (Becker 1969; Noirot and NoirotTimotheé 1969; Lee and Wood 1971; La Fage and Nutting 1978; Bustamante and Martius 1998).

Estimates of the consumption of plant necromass have demonstrated that termites are important elements in the dynamic processes of decomposition and nutrient cycling (Adamson 1943; La Fage and Nutting 1978; Abe and Matsumoto 1979; Martius 1994). In different tropical ecosystems, these insects can consume from 14 to 50% of the annual production of plant necromass (Matsumoto and Abe 1979; Holt 1987; Bignell and Eggleton 2000). In some deserts, termites can consume up to 100% of the plant necromass produced (Bodine and Uecker 1975; Whitford 1991).

In order to understand the functional role of termites in the ecological processes of decomposition and nutrient recycling, it is necessary to study biomass production and the consumption rates of a given species (or assemblage) of termites in a given environment. Quantitative data on termite populations in Neotropical ecosystems are scarce (Martius 1994), and measurements of their consumption rates in the field are quite difficult, — in large part because of the cryptic foraging behavior of a majority of the species (Wood 1978; Matsumoto and Abe 1979).

The purpose of this paper is to evaluate the participation of the populations of *Nasutitermes corniger* (Motschulsky) (Isoptera: Termitidae), *N. ephratae* (Holmgren), and *N. macrocephalus* (Silvestri) in the consumption of the plant necromass produced in a remnant area of Atlantic Coastal Forest in northeastern Brazil. These species of *Nasutitermes* are among the principal termites that construct their nests in trees, and they can be readily encountered inside decomposing tree trunks in areas of Atlantic Coastal Forest in northeastern Brazil (Vasconcellos et al. 2005; Vasconcellos et al. 2008).

## Materials and Methods

The study was undertaken in the Horto Dois Irmãos State Park, PHDI (8° 02′ S; 34° 54′ W), Pernambuco State, northeastern Brazil. The PHDI occupied an area of 387 ha, with altitudes varying from 30 to 90 MASL. The soils were nutrient-poor yellow podzols, with a predominantly sandy texture. The annual average temperature and relative humidity was 26° C and 82%, respectively. The average annual precipitation was 1824 ± 301 mm (Sampaio et al. 1988).

### Estimates of *Nasutitermes* spp. abundance and biomass

The abundance and biomass of the populations of *N. corniger, N. ephratae*, and *N. macrocephalus* were estimated in their two principal micro-habitats: nests and wood.

Nest density was estimated in ten 100 × 10 m randomly established plots in the PHDI. All of the nests encountered in these plots were counted and their volumes estimated. The termite populations inside at least four nests were quantified for each of the three species. These nests were first measured to determine their approximate volume and then opened; three fragments were then removed and weighed and the termites extracted. These fragments represented 2–15% of the total weight of the nests, and they were removed in the form of wedges from the basal, median, and upper parts of each nest. Based on the quantitative averages of the numbers of termites present in these fragments, the total population of each nest was estimated. By uniting the data of the average population per nest with the nest densities in the sampling plots, it was possible to estimate the abundance and biomass of termites per hectare in this micro-habitat.

The density of termites in wood was estimated in twenty 6 × 5 m plots laid out in the PHDI (10 in the rainy season and 10 in the dry season). All of the fallen wood larger than 1 cm in diameter was collected and weighed. Subsequently, a 5 kg sample of the wood in each plot was taken (using a chain-saw when necessary), and all of the termites in their interior were manually collected. The total abundance of termites in the wood encountered in the plot was calculated proportionally from the 5 kg wood sample.

### Quantification of wood consumption in the laboratory

Wood consumption of the three *Nasutitermes* species was evaluated using three tree species: *Clitoria fairchildiana* Howard (Fabales: Fabaceae), cut both three months and approximately four years earlier, *Cecropia* sp.(Urticales: Cecropiaceae) cut approximately two years earlier, and *Protium heptaphyllum* (Aublet) Marchand (Sapindales: Burseraceae) cut approximately six months earlier. These tree species are common in the area where the termites were collected for the experiment. The different stages of decomposition were used to represent the natural variation of wood in the field. The wood was made available to the termites in the form of small blocks measuring 2 × 2 × 1 cm. These blocks were dried at 105° C for 72 hours and re-humidified with distilled water shortly before being offered to the insects.

Sub-colonies of each termite species were held in 1.5 dm^3^ non-toxic closed plastic receptacles with a substrate of 2 cm of sterilized sand covered by 1 cm of expanded vermiculite, which has the capacity to retain water and prevent the drying of the receptacles and blocks of wood (Lenz et al. 1976). The sub-colonies were composed of 200 workers and 50 soldiers, a ratio approximating that observed in adult colonies of *Nasutitermes* spp. in an area of Atlantic Coastal Forest (Vasconcellos and Bandeira 2000). The sub-colonies in the plastic receptacles received a block of each type of wood (totaling four blocks) and were subsequently maintained in the laboratory in total darkness for 20 days. The temperature of the receptacles was maintained at approximately 26° C. For each species of termite, there were 20 repetitions of the experiment, i.e., 20 receptacles were used containing a sub-colony and a block of each type of wood. Eight receptacles with blocks of wood, but no termites, were used as controls. At the end of 20 days of consumption, the receptacles with more than 10% termite mortality were not considered for the estimates of consumption of wood.

Wood consumption was calculated as the difference between the initial and final weight of the blocks (corrected, when necessary, by the weight loss of the controls) (Bustamante 1993; Vasconcellos and Bandeira 2000). The consumption values obtained in each plastic receptacle were transformed to mg of dry wood consumed/g living termites/day. The overall participation of the species *N. corniger, N. ephratae*, and *N. macrocephalus* in the consumption of the wood litter produced at the PHDI was estimated from the results of a study of the annual litter fall in the park (Sampaio et al. 1988).

## Results

The population estimates indicated that the average abundance of the three termite species varied between 40.8 and 462.2 individuals/m^2^, with *N. corniger* being present in the largest numbers (Table 1). The nests of *N. macrocephalus* were the most highly populated (with an estimated maximum population of 803,600 individuals) while the nests themselves had volumes greater than 280 dm^3^. The nest densities of *N. corniger* and *N. ephratae* were greater than those of *N. macrocephalus* (the latter species having less than 1 nest/ha) (Table 1). The proportion of the abundance of termites in wood to the abundance in nests was 23.7/76.3, 45.3/54.7, and 0/100, for *N. corniger, N. ephratae*, and *N. macrocephalus* respectively.

**Table 1.**
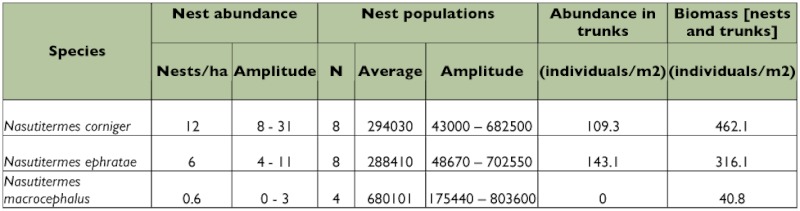
Biomass and abundance of *Nasutitermes corniger, Nasutitermes ephratae*, and *Nasutitermes macrocephalus* in their two principal microhabitats (nests and trunks) in an area of Atlantic Coastal Forest located in Pernambuco State, northeastern Brazil.

The test-blocks of *C. fairchildiana* wood in decomposition for approximately four years were the most consumed by the three termite species (Table 2). The greatest average total estimated consumption was by *N. macrocephalus* (12.1 mg of wood/g termite (fresh weight)/day), followed by *N. corniger* and *N. ephratae*, which had average consumption of 9.3 and 6.9 mg of wood/g termite (fresh weight)/day, respectively (Table 2). The average total wood consumption for the three termite species was 9.4 mg wood/g termite (fresh weight)/day.

Total wood litter consumption by the three species of *Nasutitermes* in the PHDI was estimated to be 66.9 kg wood (dry weight)/ha/year (or 6.7 g of dry wood /m^2^/year). The average estimated consumption was 4.02, 2.01, and 0.65 g of dry wood /m^2^/year for *N. corniger, N ephratae*, and *N. macrocephalus*, respectively. The data presented by Sampaio et al. (1988) for the annual production of necromass in the PHDI was used to estimate the participation of each of the three species of *Nasutitermes* in removing the wood necromass produced every year, yielding: 1.74% for *N. corniger*, 0.87% for *N. ephratae*, and 0.28% for *N. macrocephalus.*

## Discussion

Eighteen species of wood-feeding termites constitute the dominant feeding group in the area of Atlantic Coastal Forest where this study was carried out (Vasconcellos 2003). The tree-nests of *N. corniger, N. ephratae*, and *N. macrocephalus* were relatively easy to identify and distinguish by their external morphology and placement. Although the nests of *N. macrocephalus* had larger populations, greater nest densities per area, as well as total termite biomasses were recorded for *N. corniger* and *N. ephratae.* The nest densities of *N. corniger* and *N. ephratae* in the Amazon Forest were estimated to be 2.7 and 47.1 nests/ha, respectively; *N. macrocephalus* had 4 nests/ha and populations between 150,000 and 1,500,000 individuals/nest in the same area (Martius 1994).

The estimated consumption values of *N. macrocephalus, N. corniger*, and *N. ephratae* were within the amplitude range reported for other termite species under laboratory conditions (between 2.0 and 90.8 mg of food/g of termite/day) (Wood 1978). The consumption rates encountered in this study were similar to those reported for other species of *Nasutitermes* under laboratory conditions: 10.6 and 12.2 mg of food/g of termite/day for *N. exitiosus* (Gay et al. 1955; Lee and Wood 1971) and 9.0 mg of food/g of termite/day for *N. ripperti* (Hrdý and Zelaný 1967). Hrdý and Zelaný (1967) reported a consumption rate of 2.0 mg of food/g of termite/day for *N. costalis.* As this species was recently synonymized with *N. corniger* by Scheffrahn et al. (2005), this consumption rate was considerably lower that encountered in this study. Martius (1989) estimated the consumption of *N. macrocephalus* to be 16.3 mg of food/g of termite/day under laboratory conditions.

**Table 2.**
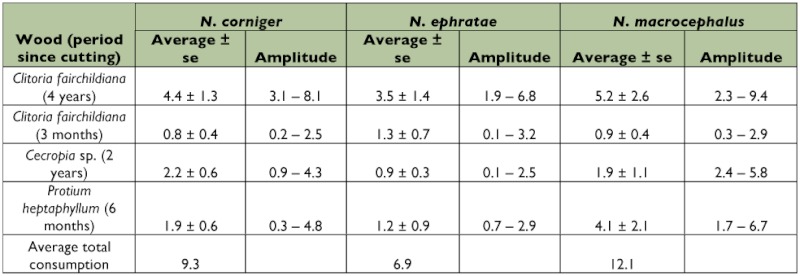
Laboratory consumption of four types of wood [mg of wood/g of termite (fresh weight)/day] by *Nasutitermes corniger, Nasutitermes ephratae*, and *Nasutitermes macrocephalus* collected in an area of Atlantic Coastal Forest located in Pernambuco State, northeastern Brazil.

Consumption rates for termite assemblages are poorly known in comparison with other ecological parameters, such as richness of species and abundance (Wood 1978; Bignell and Eggleton 2000). Field measurements are difficult to perform, whereas laboratory estimations need to control the levels of temperature and humidity and ought to prevent food decay or contamination by pathogenic organisms that may lead to abnormal mortality rates of termites (Wood 1978). In addition, the variation of food quality encountered in the field by termites is far different from the preferences recorded in laboratory experiments. Consumption rates in laboratory conditions probably reflect differences among the various species of termites, the palatability of the different species of wood offered, and the conditions under which the sub-colonies were maintained in the laboratory (Wood 1978).

The three species of *Nasutitermes* tested showed the highest rates of consumption of *C. fairchildiana* wood that had been decomposing for approximately four years. Vasconcellos and Bandeira (2000) also reported greater consumption rates for this tree species in decomposition (under laboratory conditions) by three species of termites from the Atlantic Coastal Forest, including *N. corniger.* Bustamante and Martius (1998) studied the food preferences in the laboratory of five species of *Nasutitermes* (*N. corniger, N. ephratae, N. macrocephalus, N. surinamensis*, and *N. tatarendae*) from the Central Amazon region, and likewise detected a greater preference for decomposing rather than fresh wood. Termites may prefer plant material in decomposition due to the presence of microorganisms (principally fungi) that can provide additional sources of nitrogen (Spears and Veckert 1979) and vitamins (Sands 1969), and the decomposition of the wood by these fungi probably also facilitates the mastication and degradation of cellulose compounds (Becker 1969).

Variation in wood chemistry is known to affect termite feeding (Wood 1978; Traniello and Leuthold 2000). For wood-feeding species, the nutritional value of wood may also be reflected in carbohydrate content and digestibility (Traniello and Leuthold 2000). The role of nitrogen and secondary plant compounds have also been emphasized (Abe and Higashi 1991; Traniello and Leuthold 2000). The consumption rates obtained in this study probably reflected differences in density and palatability among the tree species and different stages of decomposition.

Wood consumption by the three species of *Nasutitermes* in the PHDI was estimated to be 66.9 kg/ha/year. Sampaio et al. (1988) estimated the average annual production of necromass in the PHDI to be 11.3 tons of organic plant material/ha/year, including leaves, influorescences, fruits, branches, and twigs. The production of branches and twigs was estimated to be 1.7 and 2.9 tons/ha/year, respectively. In terms of the total necromass produced in the PHDI, the participation of the three termite species in removing this material was 0.7%). However, the three species removed between 2.9 and 3.3% of the total annual production of necromass of all the branches and twigs in the PHDI.

In the seasonally flooded (*várzea*) forests of the Central Amazon, wood consumption by *N. macrocephalus* and *N. corniger* was estimated to be 100 and 290 kg/ha/year, corresponding to 1.4 and 4.0%, respectively, of the annual production of wood (Martius 1994). Adding these figures to the consumption of the other termite species that occur in the area, it can be estimated that the wood necromass removed by termites may reach 20% of the total annual production. There is currently very little information available, however, concerning the participation of termites in the consumption of the plant necromass produced in the Neotropical region. Salicket al. (1983) estimated indirectly that the termite fauna in a forest growing on oligotrophic soils in Venezuela consumed 210 to 590 kg/ha/year of the forest necromass (corresponding to between 3 and 5% of the total necromass produced). Martius (1994) considered these values to be relatively low and attributed possible errors in these calculations to the small size of the sampling area and the consumption/biomass conversion factor used.

The participation of termites in the process of necromass decomposition in tropical forests seems less impressive than that seen in some arid and semi-arid regions. In some areas with savanna vegetation, a termite community may consume from 5% to more than 80% of the primary production (Wood and Sands 1978; Ohiagu and Wood 1979). In these ecosystems, the Macrotermitinae participate actively in the consumption of organic plant material. The importance of this subfamily and its fungal symbionts in the process of decomposition becomes even more evident when considering the high efficiency of the assimilation of material harvested and then processed in their nests, which can reach almost 100% (Wood and Sands 1978). Collins (1988) estimated the necromass consumption of a community of termites in an area of tropical humid forest in Malaysia to be between 1554 and 1732 kg/ha/year, corresponding to 14.7 to 16.3% of the total resource production. There are also species of Macrotermitinae, especially *Macrotermes carbonarius*, that participate actively in the consumption of the necromass of leaves in these forests (Matsumoto and Abe 1979).

Of the 36 species of termites that have been reported to occur in the PHDI, 18 of them belong to the group of wood-feeders (Vasconcellos 2003). Some of the species feeding exclusively on wood have high abundance and biomass in the same area of Atlantic Coastal Forest, including *Heterotermes longiceps, Nasutitermes gaigei*, and *Microcerotermes exiguus.* Although the variety of food available to termites in the natural environment was not made available in this study, wood consumption by the three species of *Nasutitermes* studied here, when summed with the consumption of the other locally occurring wood-feeding species, indicates the importance of this group of termites in removing wood litter.

## Abbreviation

PHDI: Horto Dois Irmãos State Park
